# Guillain-Barré Syndrome After Revision Lumbar Surgery: A Case Report

**DOI:** 10.7759/cureus.1393

**Published:** 2017-06-26

**Authors:** Aymen Rashid, Swamy Kurra, William Lavelle

**Affiliations:** 1 Department of Orthopedic Surgery, SUNY Upstate Medical University

**Keywords:** guillain-barré syndrome (gbs), revision lumbar spine surgery, complication

## Abstract

Guillain-Barré syndrome (GBS) is a term that is used to describe a group of immune-mediated peripheral neuropathies, with the most common feature being rapid polyradiculoneuropathy. The exact etiology of this syndrome is unknown. In the field of orthopedics, GBS has been reported to occur after total hip arthroplasty, orthopedic trauma, and spine surgery. We report a unique case of GBS after elective revision lumbar spine surgery. A 62-year-old female presented with persistent low back pain and radiculopathy and elected to have revision lumbar spine surgery. Approximately 24 to 36 hours after hospital discharge, she returned to the hospital with weakness in her legs. After an electromyography (EMG), the patient was diagnosed with GBS and placed on intravenous immunoglobulin (IVIG). She developed respiratory failure, which required intubation and eventually converted to a tracheostomy and was finally decannulated. Over the course of 12 months, she improved to her pre-surgical baseline, gaining 5/5 strength in her upper and lower extremities and was able to ambulate independently without any aids. This was a case of GBS that occurred in a patient approximately two weeks after revision lumbar surgery. GBS is a poorly understood and rare complication of lumbar spine surgery that needs to be recognized quickly to be effectively treated.

## Introduction

Guillain-Barré syndrome (GBS) is a term that is used to describe a group of immune-mediated peripheral neuropathies, with the most common feature being rapid polyradiculoneuropathy [1]. It affects approximately 0.8 to 1.9 per 100,000 people every year, and its incidence has a bimodal distribution which peaks in the young adult population (ages 20–30) and again in the elderly population (ages in the 60s) [2]. The symptoms of GBS are ascending weakness, paresthesias, and areflexia.

The exact etiology of this syndrome is unknown. Most commonly, it presents after a gastrointestinal or respiratory infection. One third of GBS cases have been reported to be associated with Campylobacter jejuni. It has also been associated with mycoplasma pneumoniae, haemophilus influenzae, cytomegalovirus, and the Epstein-Barr virus. The pathogenesis of the condition is currently thought to be multifactorial with anti-ganglioside antibodies. These antibodies attack molecules on the peripheral nerves, have molecular mimicry with infectious agents, and complement activation playing major roles [3].

It has been shown that surgery can be a trigger for GBS. A retrospective review in 2012 by Gensicke, et al. demonstrated that 9.5% of GBS cases had surgery six weeks prior and their relative risk of contracting GBS was 13.1 times higher when compared to the normal population [4]. There are case reports across various surgical fields including bariatric surgery, cancer surgery, transplant surgery, and shortly after anesthesia that may suggest that surgery could be a trigger for GBS [5-7].

In the field of orthopedics, GBS has been reported to occur after total hip arthroplasty, orthopedic trauma, and spine surgery [8]. We report a unique case of GBS after elective revision lumbar spine surgery.

## Case presentation

### History

A 62-year-old female, who had a history of chronic headaches, previous lumbar decompression and fusion at L3-4 for degenerative lumbar stenosis, presented to the clinic with low back pain. She was found to have an adjacent segment disease at L2-3 with recurrent stenosis at L3-4. The patient failed non-operative management including physical therapy and steroid injections. Consequently, she elected for surgery and underwent a revision lumbar decompression with anterior and posterior instrumentation for spinal fusion, as well as a repair for an incidental durotomy. The patient did well after surgery and was discharged home nine days later.

### Examination

Approximately 24 to 36 hours after discharge, the patient fell due to weakness in her legs. She was seen in the emergency department and was found to have progressive weakness, numbness, and areflexia in her bilateral lower extremities. Over the course of the ER visit, she developed ascending paralysis as well. She denied any fever, chills, or symptoms of infection. The initial laboratory workup was negative for infection. At that time, a magnetic resonance imaging (MRI) only showed postsurgical changes. Neurology was consulted and did not recommend a cerebrospinal fluid (CSF) sample due to the recent surgery.

Because of a clinical suspicion for GBS, she was admitted to the neurological intensive care unit (ICU) and started on intravenous immunoglobin (IVIG) therapy for six days. The patient’s hospital stay was complicated by the development of respiratory failure, which required intubation. She was eventually converted to a tracheostomy and was finally decannulated. An electromyography (EMG) showed diminished motor and sensory responses in terms of amplitude and conduction velocity in her upper extremities and absent amplitude and conduction velocities in her lower extremities confirming the diagnosis of GBS.

### Post-hospital admittance

After an approximate four-week hospital admittance, the patient was discharged to a rehabilitation facility and continued aggressive physical therapy. At the time of discharge, she had a 4+ strength in her upper extremities and a 2+ strength in her lower extremities. The patient continued with outpatient physical therapy and over the course of 12 months, she improved to her pre-surgical baseline, gaining 5/5 strength in her upper and lower extremities and was able to ambulate independently without any aids.

## Discussion

In orthopedic literature, there have been a few case reports of Guillain-Barré syndrome occurring after trauma, total hip arthroplasty, and spine surgery. Only one study reported the incidence of GBS after spine surgery, one per 2000 cases [9]. While there are case reports of GBS occurring after spine surgery, there are no reports of GBS after revision surgery. Our patient did not have GBS after her first lumbar spine surgery nor did she have any episode of GBS with her subsequent total hip arthroplasty surgeries. Based on the patient’s records, it does not appear that there were any significant differences in the preoperative, perioperatove, and postoperative care between her first surgery and this surgery.

GBS presents with a combination of rapid worsening paralysis, numbness, areflexia, and pain. The symptoms typically progress from 12 hours to 28 days after presentation, and then GBS undergoes a plateau phase for approximately one to three weeks. It typically affects the proximal muscles as well as the lower extremities more than the upper extremities. In addition to a physical examination, GBS is usually diagnosed either with a cerebral spinal fluid (CSF) analysis or EMG. The CSF analysis shows typical albuminocytologic dissociation with elevated protein without pleocytosis and an EMG shows absent or diminished nerve potentials.

The link between surgery and GBS is not well understood. The initial presentation of GBS varies among case reports with some patients having symptoms within hours of surgery and others, as in this case, having symptoms within weeks of surgery. Initially, it was thought that T-lymphocyte activation was the primary mechanism. It has also been shown that antimyelin T-cell injection results in GBS in animals. However, there is no good evidence to support that the same mechanism occurs in humans. One postulated mechanism is the surgical effect on the immune system. By causing an activation of the endocrine stress systems, surgery leads to transient immunosuppression, which would allow autoantibodies to promote an attack of the peripheral nerves. This idea is promoted by the fact that patients with HIV and transplant patients on immunosuppression have triggered GBS.

The treatment of GBS is based on two components: supportive therapy and immunotherapy. Approximately 25% of patients have respiratory failure and require intubation. In addition, they can require the need for a temporary pacemaker. Immunotherapy involves either plasma exchange or the administration of intravenous immunoglobulin. Despite this, 20% of patients remain disabled and 5% of patients die.

## Conclusions

In summary, we present a case of GBS that occurred in a patient approximately two weeks after revision lumbar surgery. GBS is a poorly understood and rare complication of lumbar spine surgery that needs to be recognized quickly to be effectively treated.
